# PRECISION.seq: An R Package for Benchmarking Depth Normalization in microRNA Sequencing

**DOI:** 10.3389/fgene.2021.823431

**Published:** 2022-01-28

**Authors:** Jian Zou, Yannick Düren, Li-Xuan Qin

**Affiliations:** ^1^ Department of Biostatistics, University of Pittsburgh, Pittsburgh, PA, United States; ^2^ Department of Mathematics, Ruhr-University Bochum, Bochum, Germany; ^3^ Department of Epidemiology and Biostatistics, Memorial Sloan Kettering Cancer Center, New York, NY, United States

**Keywords:** microRNA, sequencing, normalization, benchmarking, software

## Abstract

We present a new R package *PRECISION.seq* for assessing the performance of depth normalization in microRNA sequencing data. It provides a pair of microRNA sequencing data sets for the same set of tumor samples, additional pairs of data sets simulated by re-sampling under various patterns of differential expression, and a collection of numerical and graphical tools for assessing the performance of normalization methods. Users can easily assess their chosen normalization method and compare its performance to nine methods already included in the package. *PRECISION.seq* enables an objective and systematic evaluation of normalization methods in microRNA sequencing using realistically distributed and robustly benchmarked data under a wide range of differential expression patterns. To our best knowledge, this is the first such tool available. The data sets and source code of the R package can be found at https://github.com/LXQin/PRECISION.seq.

## Introduction

Depth normalization is a critical preprocessing step for accurate and reproducible analysis of transcriptomic sequencing data (Bullard et al., 2010). Methods for depth normalization have been newly proposed or repurposed from normalization methods previously developed for microarray data (Dillies et al., 2013). Their performances have been evaluated primarily for RNA sequencing data and a thorough assessment is still in need for microRNAs (miRNAs), a class of small RNAs regulating gene expression and closely linked to carcinogenesis, which tend to be expressed in a tissue-specific manner with a small number of markers abundantly expressed (Dillies et al., 2013; Maza et al., 2013).

To enable such an assessment, we collected two data sets for the same set of tumor samples, where one set was collected using uniform handling and balanced library assignment and the second was collected over time and without such careful study design (Qin et al., 2020). The former can be used to assess miRNAs’ differential expression (DE) status, serving as a benchmark; the latter can be used to assess the use of normalization methods against the benchmark. Furthermore, we devised a re-sampling-based strategy for simulating additional data set pairs and developed a workflow for performing the paired-data-sets based assessment. We have built these data and the workflow into an R package named *PRECISION.seq*, PaiREd miCrorna analysIs of differential expresSION for sequencing, for interested researchers to assess methods.

## Implementation

MiRNAs were sequenced for 27 myxofibrosarcoma samples and 27 pleomorphic malignant fibrous histiocytoma samples twice, once with uniform handling (serving as the “benchmark” data) and a second time in the order of sample collection over the years resulting in unwanted depth variations (serving as the “test” data) (Qin et al., 2020). The first data set can be accessed by *data.benchmark* and the second by *data.test*.

The overall normalization assessment is provided by the function *precision.seq()* following three steps: first, the test data is normalized using one or multiple methods; second, differential expression between the two subtypes is determined in the un-normalized benchmark data and normalized test data using either *voom-limma* or *edgeR* (Robinson, McCarthy, and Smyth 2010; Law et al., 2014); lastly, the DE statuses determined in the benchmark data are used as a gold standard for assessing the performance of normalization methods in the test data.

Our package currently includes nine normalization methods that are relatively commonly used in the literature. Among them, six methods are based on scaling: Total Count, Upper Quartile, Median, Trimmed Mean of M-values (TMM), DESeq, PoissonSeq (Anders and Huber 2010; Robinson and Oshlack 2010; Li et al., 2012; Dillies et al., 2013); three methods are based on regression: Quantile Normalization, Surrogate Variable Analysis for Sequencing (SVASeq), and Remove Unwanted Variation (RUV, including three sub-methods RUVg, RUVr, RUVs) (Irizarry et al., 2003; Leek 2014; Risso et al., 2014). The computational speed of these normalization methods is very fast, in the range of a fraction of seconds for scaling methods and about a second for the RUV methods, using a PC with AMD Ryzen 5 3600 6-Core Processor 3.60 GHz. Users can also add any additional normalization method to the workflow by providing its normalized test data to the *precision.seq()* function.

The differential expression analysis results are compared numerically and graphically between the normalized test data and the un-normalized benchmark data. Treating the latter as a gold standard and dichotomizing the *p*-values at a user-specified significance level, the *pip.statistics()* function calculates the True Positive Rate (TPR), False Positive Rate (FPR), False Discovery Rate (FDR), and False Negative Rate (FNR). To assess the impact of each individual normalization method, functions are included to draw 1) Relative Log Expression (RLE) plot for log2 count data (*fig.RLE*()), 2) Volcano plot for *p*-values versus group mean differences (*fig.volcano*()), and 3) Venn diagram of DE statuses (*fig.venn*()) (Gandolfo and Speed 2018). To compare across normalization methods, functions are provided to draw 1) Scatter plot of FNRs and FDRs (*fig.FDR_FNR*()), 2) Concordance At the Top (CAT) plot of the *p*-value ranking (*fig.CAT*()), and 3) Dendrogram for hierarchically clustering *p*-values using the Euclidean distance and the Ward’s minimum variance linkage (*fig.dendrogram*()) (Waldron et al., 2012).

Additional paired data sets can be simulated under various scenarios of differential expression using the *simulation.algorithm*() function that implements the re-sampling-based algorithm introduced in (Qin et al., 2020). Briefly, sample group labels are shuffled for the benchmark data by: 1) clustering the 54 samples to two clusters and randomly selecting nine samples in each cluster to serve as the ‘anchor samples’ for the two new sample groups; 2) randomly allocating the remaining 36 samples to these two new sample groups. The same sample shuffling is then applied to the test data. We have used this algorithm to pre-simulate 20,000 pairs of data sets and categorized them based on the proportion of differential expression and the median of mean differences across markers. To save computation time, users can extract the pre-simulated data sets under a desired differential expression pattern using the *simulated.data()* function. The simulated data sets can be analyzed by calling the function *pip.simulated.data()* and the results can be summarized and displayed by calling the *fig.FDR_FNR.boxplot()* function.

## Example Usage

**FIGURE 1:**
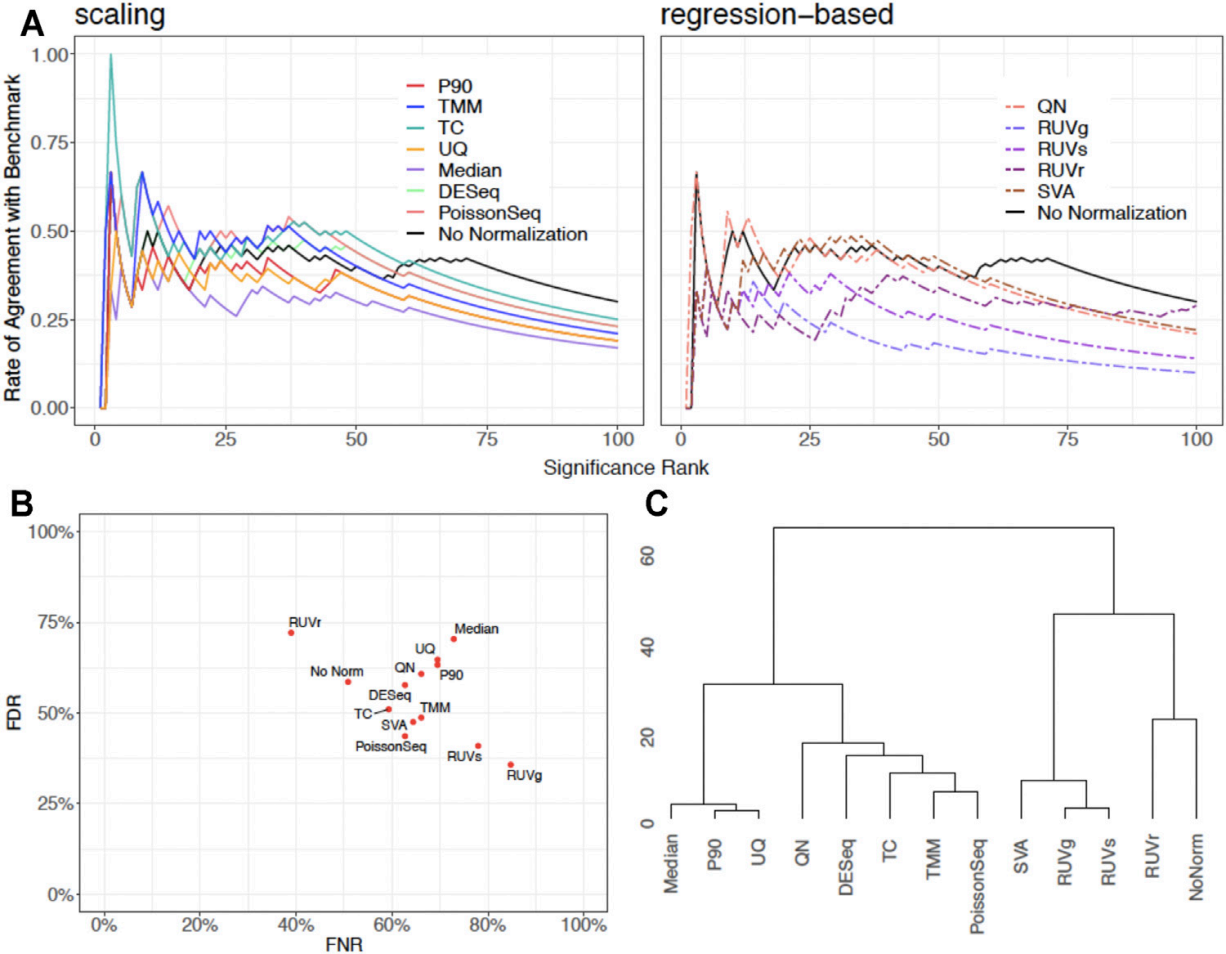
Graphical display of empirical assessment results for P90 normalization in comparison with no normalization and nine normalization methods using *PRECISION.seq*. **(A)** CATplot of *p*-value ranking determined in the test data that undergoes scaling normalization (left panel) or regression-based normalization (right panel), in comparison with no normalization. **(B)** Scatterplot of False Negative Rate and False Discovery Rate among the normalization methods. **(C)** Dendrogram for clustering the *p*-values in the test data before and after normalization.

**FIGURE 2:**
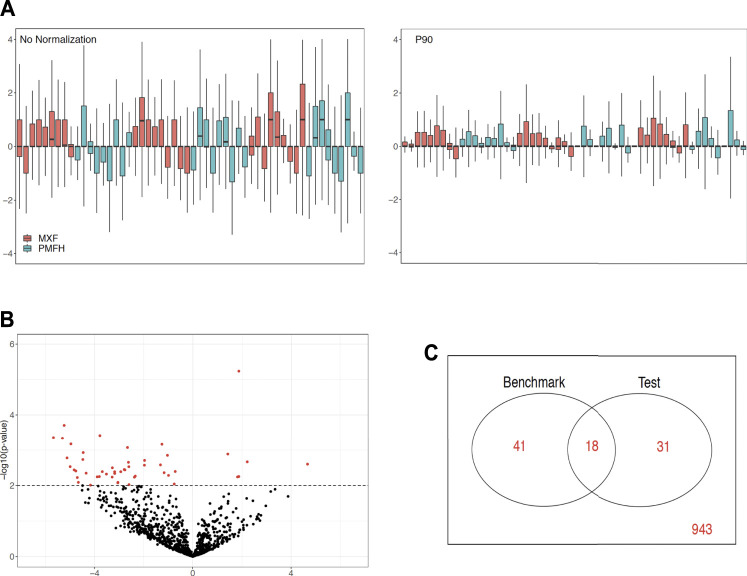
Graphical display of method-specific empirical assessment results for P90 normalization using *PRECISION.seq*. **(A)** RLE plot for log2 count data before (left panel) and after P90 (right panel), **(B)** Volcano plot for *p*-values versus group mean differences after P90, **(C)** Venn diagram of DE statuses before versus after P90 normalization.

**FIGURE 3:**
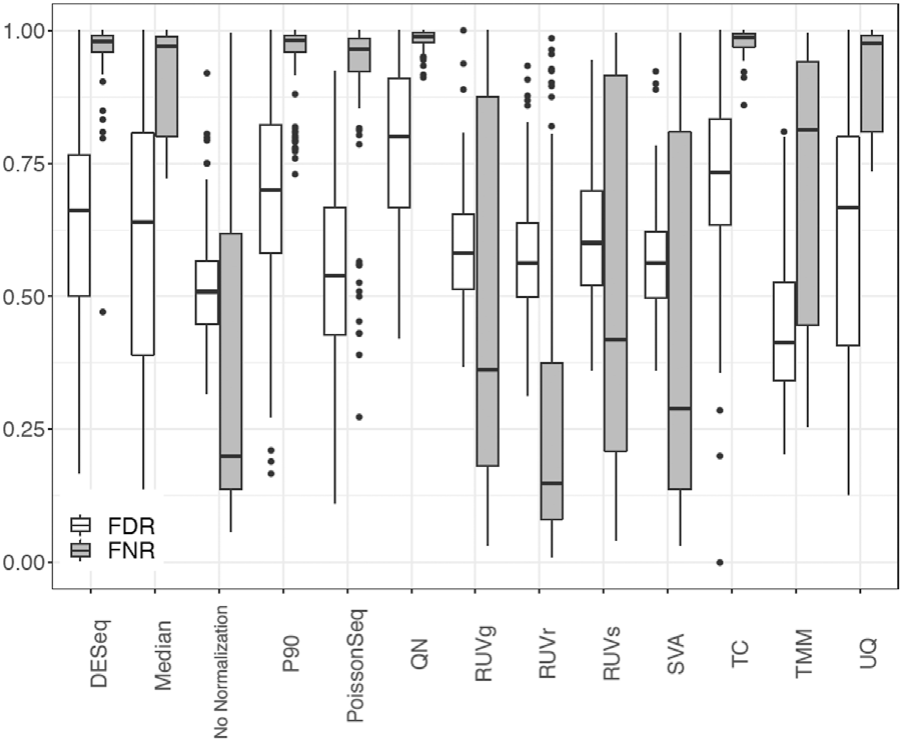
Boxplot of FDR and FNR in 100 pairs of simulated data sets for P90 normalization in comparison with no normalization and nine normalization methods using PRECISION. seq.

We showcase the use of the benchmarking pipeline for scaling normalization by the 90th percentile (P90). We assess the performance of P90 in comparison with the nine aforementioned methods using the function *precision.seq()*. Assessment is first done with the pair of empirical data sets (Figure 1). As expected, P90 performs similarly to Upper Quartile and Median Normalization due to their related manner of normalizing the data. More specifically, P90 is a moderate performer resembling Upper Quartile and Median Normalization in terms of *p*-value ranking; it has an FDR of 63.27% and an FNR of 69.49%; its *p*-values cluster closely with those for Upper Quartile and Median. Method-specific plots for P90 are provided in Figure 2. Further assessment is done using 100 pairs of simulated data sets that have a DE proportion around 20% and a median of mean differences around 3. P90 shows mediocre FDR and poor FNR, generally comparable to Total Count and Quantile Normalization and slightly worse than Upper Quartile and Median (Figure 3).

## Summary

In this paper, we introduce an R package, called *PRECISION.seq*, for assessing the performance of depth normalization methods in miRNA sequencing using realistically distributed and robustly benchmarked data under a range of differential expression scenarios. To the best of our knowledge, this is the first such tool available. One limitation of our tool is that it does not offer varied scenarios for the number of samples or the range of sequencing depth in the samples.

## Data Availability

Publicly available datasets were analyzed in this study. This data can be found here: https://github.com/LXQin/PRECISION.seq.DATA.
